# Commercial mHealth Apps and Unjust Value Trade-offs: A Public Health Perspective

**DOI:** 10.1093/phe/phac016

**Published:** 2022-09-02

**Authors:** Leon W S Rossmaier

**Affiliations:** The University of Twente, Enschede, The Netherlands

## Abstract

Mobile health (mHealth) apps for self-monitoring increasingly gain relevance for public health. As a mobile technology, they promote individual participation in health monitoring with the aim of disease prevention and the mitigation of health risks. In this paper, I argue that users of mHealth apps must engage in value trade-offs concerning their fundamental dimensions of well-being when using mobile health apps for the self-monitoring of health parameters. I particularly focus on trade-offs regarding the user’s self-determination as well as their capacity to form personal attachments. Depending on the user’s level of advantage or disadvantage, value trade-offs can pose a threat to the users’ sufficient fulfillment of the dimensions of well-being. As such, value trade-offs can entrench existing structural injustices and prevent disadvantaged users to benefit from this technology. I argue that value trade-offs are, to some, a type of injustice that can drive disadvantaged users away from a sufficiency threshold of well-being, risk users to fall below the threshold, or have an accumulative effect on different dimensions of the user’s well-being.

## Introduction

mHealth apps bear great potential for promoting public health. A widely used definition by the WHO states that: ‘[mHealth is a]…medical and public health practice supported by mobile devices, such as mobile phones, patient monitoring devices, personal digital assistants (PDAs), and other wireless devices’ (World Health Organization, 2011, p. 6). The opportunities of this technological domain are widely recognized by international public health organizations like the WHO and the European Commission (World Health Organization, 2011; European Commission, 2014) as well as policymakers who emphasize its importance for national and regional health systems in rich and poor countries alike (Barkman and Weinehall, 2017).

As consumer products, commercial mHealth apps present the possibility for users to self-manage, measure and monitor their health to support or initiate behavior change. In contrast to non-commercial mHealth apps, commercial mHealth apps often entail a lucrative business model based on the collection and analysis of digital health-related data. The different functionalities of mHealth apps vary broadly since apps with new functions enter the market every day. The focus of this paper lies on apps that enable the self-monitoring and measuring of the users’ health in almost every life situation. It is this aspect of mHealth apps that distinguishes them from other forms of telemedical products and services.

In most cases, commercial as well as non-commercial mHealth apps are accessible via the Google or Apple app stores. A clear categorization of mHealth apps is often difficult since they are usually not classified as medical products while their data can be relevant for professional treatments and diagnosis. Examples of commercial mHealth products for self-monitoring are apps for period tracking, diet tracking, exercising or the self-management of chronic diseases that entail a data-centered business model. Aside from the technical prerequisites of the users’ smartphones or tablets the use of mHealth apps is often supported by portable sensors like smartwatches or fitness bracelets, enabling the increasingly precise recording of body and health-related data.

Public health, as not only being a top-down measure but a collective participatory effort (Verweij and Dawson, 2007) is supported by commercial mHealth apps due to their increasingly important role in primary care (Bally and Cesuroglu, 2020). The reasons for this being wider, easier and cheaper access to health services (Lucivero and Jongsma, 2017), their role in preventative measures (Burke *et al.*, 2015; Albrecht, 2016), as well as their potential for ubiquitous data-driven health monitoring and personalized care (Cvrkel, 2018). Especially mHealth apps for self-monitoring and self-management of health support the behavioral turn in public health that increasingly focuses on individual behavior changes toward a healthier lifestyle as a strategy to respond to major public health challenges like diabetes, pulmonary and cardiovascular disease (Brown, 2018). This approach promises to enable patients to become more self-determined by making them increasingly responsible for their own health and well-being. Although this personal responsibilization has been generally criticized for demanding unacceptably comprehensive forms of surveillance (Davies, 2021), the question persists if the downsides of using mHealth apps, as well as the benefits, have equal moral weight for all members of society.

Given the relevance of mHealth apps for public health, the question arises of how it affects differently situated groups. Presupposing distinct positions of privilege and disadvantage, I am going to provide an approach for determining whether the use of mHealth apps entrenches existing structural injustices, or in other words unjust patterns of disadvantage, within the area of public health.

My question is motivated by the ethical debate on mHealth that has only paid little attention to the implications of mHealth for structural injustice (Herzog *et al.*, 2022), particularly in the domain of public health (Sauerborn *et al.*, 2022). To answer this question, I am going to draw from the normative framework for structural injustice by Madison Powers and Ruth Faden (Powers and Faden, 2006, 2019). This approach argues that patterns of disadvantage result in, and can result from, impairments of a person’s well-being in the dimensions of health, equal respect, self-determination, personal attachment, personal security and knowledge and understanding. Mitigating those impairments is the objective of public health (Powers and Faden, 2006). The approach enables us to view structural injustices through the lens of multiple dimensions of well-being and opens the space of ethical reflection on mobile health apps to a broader scope of criteria beyond their impact on the users’ health. If the implementation of mHealth apps increases—or risks to increase—the level of disadvantage for disadvantaged persons in the sense that it leads to decreases in the core dimensions of their well-being to the level of insufficiency, then the implementation of mHealth apps does not only pose moral challenges but also stands in the way of promoting public health.

I will start by arguing that users of mHealth apps must engage in certain value trade-offs affecting their self-determination as well as their capacity to form personal attachments. Value trade-offs refer to instances in which users of mHealth apps trade off an improvement in one dimension of their well-being, like self-determination, against an improvement or expected improvement in another dimension, like health.

Value trade-offs have been prominently discussed at the engineering level and the design for specific values in the context of conflicting values in design decisions (Friedman *et al.*, 2008; Van de Poel, 2015). My focus, however, lies in the trade-offs required by the users. In this paper, I argue that the implications of the trade-offs are of different severity according to the level of privilege or disadvantage of the users. While value-sensitive design can certainly contribute to mitigating those implications (Jacobs, 2020a), it is limited in its ability to address the broader social situation of individuals or groups and the preconditions of the initial use of mHealth.

Disadvantaged users might enter a position where they increasingly deviate from the sufficient realization of the fundamental dimensions of their well-being or risk deviating from this threshold, which stands in contrast to the overall goal of public health. My presented argumentation concludes that value trade-offs concerning the different dimensions of well-being are especially severe for persons who are already subject to the insufficient fulfillment of various dimensions of their well-being while such trade-offs are less problematic for persons who sufficiently reach this requirement. Even independently from their level of advantage or disadvantage, users of mHealth apps might want to avoid a position in which they need to give up on something that is of value to them, like the interaction with a physician or a certain level of self-determination.

## Structural Injustice

Madison Powers and Ruth Faden identify the sufficient fulfillment of six core dimensions of well-being—health, equal respect, self-determination, personal attachment, personal security and knowledge and understanding—as the necessary conditions for social justice as well as the primary objective of public health measures (Powers and Faden, 2006, 2019). They argue that those dimensions should function as a rough measurement to determine the legitimacy of institutional and organizational action because the core dimensions express a set of interests most people would agree on. Rather than understanding the core dimensions of well-being as requirements for a good life, they constitute desirable circumstances that should not be undermined but promoted by public and private organizations (Powers and Faden, 2019, p. 54). This requirement also applies to mHealth as a public health measure as well as its implementation by either public or private agents such as hospitals, software development companies, or health insurance companies.

Powers and Faden understand disadvantage as a circumstance that ‘occurs whenever any social structural impediment […] diminishes an individual’s most important well-being prospects, or in other words her prospects for a decent human life’ (Powers and Faden, 2019, p. 16). I focus on an understanding of structural injustice as unfair patterns of disadvantage and privilege. Such patterns result from unfair distributions of power in social practices that contribute to the deprivation of well-being (Powers and Faden, 2019, p. 1) for people who may belong to marginalized groups because of their gender, ethnicity, age, physical appearance, etc.

In the context of mHealth apps and other well-being technologies, different types of structural injustices have been pointed out. Especially apps targeted at users identifying as women focusing on women’s health have been criticized for promoting gender stereotypes and showing sexist and heterosexist attitudes (Hendl *et al.*, 2019; Hendl and Jansky, 2021). Deborah Lupton has argued that developers of mHealth apps often ignore differences in the socio-economic position of their users resulting in power asymmetries within healthcare (Lupton, 2018), and other scholars have pointed out the role of digital inequalities like low digital competency and access to the internet (Paldan *et al.*, 2018). Powers and Faden, however, present a theory that claims to address all structural injustices by identifying them as unjust deprivations of the core elements of well-being. It thus opens the space for a broader discussion on the relationship between the use and implementation of mHealth apps and structural injustices.

In contrast to other sufficiency theories of social justice, like the capabilities approach developed by Amartya Sen (Sen, 2011) and Martha Nussbaum (M. C. Nussbaum, 2000), Powers and Faden focus only on the functionings eventually realized for each individual, rather than the capabilities to bring about those conditions. The motivation for this view roots in the emphasis on the realized outcomes of public health interventions improving social justice for populations who are the victims of clustering disadvantages. What counts for them, are not abstract capabilities, rights or opportunities, but realized conditions showing in improvements of well-being.

Jonathan Wolff and Avner De-Shalit deliver the empirical foundation for the claim that functionings like health, personal attachment and self-determination belong, among others, to the aspects of human life that need to be fulfilled sufficiently for every member of society (Wolff and De-Shalit, 2007). Although they abstain from claiming that those functionings objectively compose a good human life, those values are central to a decent life, whether in the form of conditions for well-being or in the form of criteria for measuring disadvantage. Although the definitions of central values for a decent human life differ between theories, they coherently overlap in the cases of both approaches.

Basing the analysis of the implications of mHealth apps for disadvantaged users on the frameworks by Powers and Faden as well as Wolff and De-Shalit not only widens the theoretical scope of the ethical evaluation but also points out a way of approaching the impacts of mHealth use on disadvantaged users by empirical means. Focusing on dimensions of well-being allows us to see the benefits and downsides of the use of mHealth apps more precisely as well as their interaction with their users’ life contexts.

## The Use of mHealth Apps and Value Trade-offs

mHealth apps seem to present users with a choice. Users can either accept the terms and conditions the provider sets out for them as well as the design, the features and the business model of the app or they can abstain from using the app entirely. While such a mechanism is not inherently undesirable in other contexts, in the case of mHealth apps it runs the risk of undermining some of the fundamental conditions for human well-being and thus gains normative relevance. It seems that users must accept limitations or risks to conditions of their well-being, like self-determination or personal attachment, to improve another important condition to their well-being—health.

Central to this paper are value trade-offs concerning health on the one side and self-determination and/or personal attachment on the other side. Both values are only examples illustrating how such trade-offs can impact the users’ well-being to the extent of increasing their disadvantaged situation. Moreover, they are central to frequently mentioned ethical concerns about the risks of mHealth apps to their users’ autonomy (Burr *et al.*, 2020) and the potential to increase isolation (Eccles, 2015). Although there do exist other trade-offs, users must engage in, the focus on self-determination and personal attachment is sufficient to show the moral dimensions of such trade-offs and provides paradigmatic cases for showing that deprivations in both dimensions of well-being can have an accumulative effect on the users’ level of disadvantage.

The effects of mHealth interventions on the users’ health are highly context-dependent. While mHealth can serve as a serious intervention for promoting health by helping to self-manage chronic illnesses like diabetes (Kitsiou *et al.*, 2017; Mayberry *et al.*, 2019), the positive effect on the users’ health cannot clearly be determined or is rather marginal in other areas, like in changing the users’ diet and eating habits (McCarroll *et al.*, 2017). However marginal the positive effect on the users’ health may be, I will assume that the users’ health will indeed benefit from engaging with a mHealth app, or that they can at least reasonably expect a health benefit.

An obstacle to this claim is that developers often intentionally leave the conception of health inherent to mHealth apps unclear. Marijn Sax even identifies the arbitrary conceptualization of health in mHealth apps as the prime driver for binding users to such products to capitalize on the users’ interaction with the product (Sax, 2021). Especially mHealth interventions used by disadvantaged persons can, however, make all the difference since they provide access to health services that are otherwise not accessible to them (McBride *et al.*, 2018; Saleh *et al.*, 2018). This might be the case for migrants lacking the necessary language skills of their country of residence or persons who must rely on such products because they live in an area with poor medical infrastructure.

To illustrate how users of mHealth apps must engage in certain trade-offs, I am going to use the following examples: First, consider the case of Jenny. Jenny is a 17-year-old teenager. Like many of her peers, she uses social media platforms to communicate with her friends and family, upload pictures of herself, and follow-up with her interests. Through social media, she is vastly exposed to a permanent feed of pictures of idealized body types which results in a constant feeling of dissatisfaction with her own body and physical appearance. The negative effects of social media use especially on young women’s body images is widely documented (Casale *et al.*, 2021). Jenny follows social media accounts by female influencers who display their seemingly perfect bodies while promoting healthy eating habits and a strict exercise routine. To mitigate the dissatisfaction with her own body and lifestyle, which she perceives as unhealthy, Jenny decides to start exercising. For this, she buys a fitness bracelet by Fitbit^®^ and installs the company’s app on her smartphone. Fitbit^®^ advertises its products with the promise to enable its users to achieve their personal goals, no matter how ambitious, as well as to lead a self-determined, healthy lifestyle (Fitbit, 2021).

The app together with the wearable allows Jenny to monitor her daily activity, exercise regime, heart frequency, burnt calories, sleeping patterns and diet (Fitbit, 2021). She can share the results of her training with other users and compare herself in competitions. Because Jenny is generally insecure about her body, she feels the need to invest a lot of time and energy. She trusts the app’s incentives to exercise reaching her through regular notifications and reminders. Whenever she does not exercise and misses to follow the app’s plan, a feeling of inadequacy and guilt overcomes her. She starts punishing herself for not exercising by eating less. Although Jenny has not been diagnosed with an eating disorder or anorexia she is certainly at risk since the use of fitness apps correlates with increases in distorted eating and exercising behavior (Hahn *et al.*, 2022). Being a young woman, Jenny is generally more at risk of developing an eating disorder due to the use of fitness apps than members of other demographic groups (Hahn *et al.*, 2021).

Naomi Jacobs argues that users with eating disorders are particularly vulnerable to the incentive structures inherent to the design of fitness apps. This vulnerability consists of the inability to stop using the app and becoming increasingly dependent on the practice of self-measurement (Jacobs, 2020b). It certainly is the case that for people who are generally confident with their bodies using a fitness app might be a valuable way to reach their fitness goal and lead a healthier and more active lifestyle. For others, like Jenny, it can pose a threat to mental and ultimately physical health.

In the second case, the mHealth user’s name is John. John is 48 years old, obese, and suffers from diabetes type II. Because of his obesity, he was often bullied in school as a child which makes it hard for him to trust others and form friendships. Living on his own and being increasingly limited in his mobility he is often socially isolated and lonely. He seldom leaves his apartment and relies on delivery services for groceries and medications. Out of convenience, John downloads an app for the self-management of his chronic condition.

The mySugr app allows him to track his blood glucose, blood pressure, carbohydrate intake, diet and exercise activity. John can share this data with his doctor digitally instead of visiting in person. The provider of the app promises users an increased level of self-management and comfort regarding the collection and analysis of data related to their illness and the management and dosing of medication (mySugr, 2022).

After a while, using the app becomes an established substitute for his in-person check-ups. He keeps track of his illness and uses the app to calculate his insulin doses. Every now and then he sends data from the app to his doctor instead of visiting in person. Visiting his doctor, who has known John for years, was one of the few opportunities to engage and chat with a person face to face and discuss health and well-being issues on a broader scale.

Being already isolated any impairment on John’s personal attachments worsens his prospects of being embedded in social relationships. He traded off personal attachment to some extent for the app’s promised health benefit. Although the impairment on his social life might seem insignificant, the app affects him in a way in which he cannot quite benefit as much as somebody else would. Diabetes apps can certainly be a great and valuable tool for somebody who is tied into tight social relationships. John, however, although perhaps gaining some independence, needs to accept other deprivations to his well-being rooted in his disposition.

Both Jenny and John start using mHealth products from a vulnerable position linked to circumstances that are out of their control but continue to impact their lives and well-being in an undesirable way. Instead of focusing on the concept of vulnerability, I want to elaborate on how patterns of disadvantage that I see as the root of many vulnerabilities are affected by the use of mobile health apps. I want to stress that such vulnerabilities especially occur in specific social groups. In the case of Jenny, the risk of developing an eating disorder is highest among young women, while in the case of John, a higher risk for developing obesity correlates with lower education and low socioeconomic status (Gensthaler *et al.*, 2022). It is thus relevant to see the vulnerable position from which a user starts engaging with a mHealth app within the bigger picture of patterns of disadvantage. I specifically want to draw attention to the question of how deprivations within core elements of well-being, like self-determination or personal attachment, that are linked to mHealth, result in increased disadvantages for those who already face such deprivations.

## Self-determination

Powers and Faden understand self-determination as a necessary condition for human flourishing allowing an individual to shape the course of their life according to their own life plan (Powers and Faden, 2006, p. 27). Wolff and De-Shalit identify loss of control over one’s environment as one of the categories of disadvantages (Wolff and De-Shalit, 2007, p. 56). For them, this includes perceiving oneself as having an impact on the factors determining one’s life. Both notions of control over one’s life are compatible because they share the same objective: being able to realize one’s own life goals.

The conceptualization of self-determination as control over the course of one’s life limits the capacity to understand how self-determination is restricted through the use of mHealth apps. While the course of a person’s life is certainly relevant in the context of structural injustices, the evaluation of restrictions to self-determination needs to start at a point closer to the point of interaction between the user and the app. I, therefore, suggest, extending the notion of self-determination put forward by Powers and Faden as well as Wolff and De-Shalit by the view that self-determination is closely linked to the identity of a person. This two-pronged approach to self-determination allows for an analysis starting at the micro level; i.e. the interaction of the user with the app, and showing the consequences of this interaction for the broader context of the user’s life.

Self-constituting views on self-determination claim that individuals self-constitute by the choices they make, the goals they endorse, as well as the values they cherish (Vugts *et al.*, 2020, p. 11). To harm self-determination thus not only occurs due to coercion and the restriction of options a person has but also by making persons endorse ideas and values that are not authentic to them. In other words, to indoctrinate them with ideas and values that are not their own.

Users of mHealth apps must engage in trade-offs in which they may compromise their self-determination to gain a certain health benefit. Self-determination is compromised due to the normative conception of health inherent to mHealth apps (Morley and Floridi, 2019). The normative conception of health in mHealth apps results from the design and functioning of the products. mHealth apps designed for the tracking of health parameters operate in a way so only quantifiable aspects of a person’s health are recorded by the app. The decision on which parameters to acquire depends on the developers of the app as well as the sensors and technical capacities of the device on which it is installed. Collecting data about a certain phenomenon is always a selective task based on subjective observations and choices (boyd and Crawford, 2012). It necessarily includes a notion of what *should* be of interest and therefore entails a normative judgment reflected in the data. Furthermore, data only acquires meaning within a certain context. For us to make sense of it, it needs to be interpreted and thus, although it may seem like objective knowledge, can never live up to this expectation (boyd and Crawford, 2012). Users of a mHealth app might, however, perceive the collected data as a comprehensive and objective image of their health status, if the app records the data correctly, thus trusting the app and any recommendations it proposes. There is certainly a risk that the user could be convinced of health-related information displayed by the app that stands in conflict with their health since apps can only consider quantifiable aspects of health and may neglect other qualitative dimensions, like those relevant to mental health or well-being more generally.

Jessica Morley and Luciano Floridi point out this aspect of mHealth by referring to it as the digital medical gaze (Morley and Floridi, 2019). Using mHealth technology, the user is separated into various data flows forming a transparent data persona, or digital self. The use of digital health tools serves the user as a point of self-reflection. The user refers to the digital persona to determine whether a professional health worker or practitioner would perceive it as healthy or unhealthy. This interrogation acts as the motivation for health interventions by which the user tries to shift toward this projected notion of health. In other words, the user is motivated to act upon this perception, rather than relying on their own intuition. The reflective scope the user can achieve through the digital medical gaze is restricted by the data the mHealth app collects and displays. The design of the app thus determines how the users perceive themselves as healthy—or for that matter unhealthy—persons. However, it is not only relevant how the users perceive themselves. This self-reflection also entails a motivation for action causing the user to live up to the expectations of the imagined health professional on their digital persona and thus creating a self-disciplining effect that impacts the user’s life on a broader scale. This circumstance is, at the core, what renders the conception of health inherent to mHealth apps normative.

This normativity, however, must be evaluated carefully since it does not necessarily limit the users’ self-determination. In fact, it offers an opportunity for the users to achieve their authentic health-related goals in more efficient and effective ways (Wagner, 2019) thereby contributing to the users’ self-determination. This effect, however, depends on the question of whether the app allows the user to align their authentic goals with the normative structure of the app.

Rebecca Brown argues that public health has experienced a behavioral turn in recent years meaning that public health interventions increasingly focus on behavior change regarding lifestyle behavior like smoking, diet, exercise, etc. Those lifestyle aspects are crucial to address in the fight against heart disease, cancer, lung disease and diabetes. Attempts to change the individuals’ behavior may, however, result in healthism which is ‘1. the tendency to view an increasing number of activities and domains of life in terms of the impact they have on health, and 2. the promotion of health to a “super-value”’ (Brown, 2018, p. 999). The moralization of health in this way may cause harm to individuals because it may misguide their beliefs about appropriate health and the measures to maintain it. Incentivizing behavior change using social networks, gamification mechanisms and other methods is a common approach in many mHealth products to realize the health goals set out for the user. The use of such products increases the uncertainty about whether the normative underpinnings of the app align with the users’ authentic wish to lead a healthy life or if they promote healthism.

Furthermore, the app may direct and frame the users’ wish for a healthier life in such a way, that it primarily serves the purpose of the app’s provider. Marijn Sax argues that the user’s autonomy is restricted by the choice architectures inherent to many mHealth apps which aim primarily at binding the users to that product or an ecosystem of different products (Sax, 2021). Developers and providers intentionally avoid setting the standard for when a user counts as healthy to promote increased engagement with the app.

The developer’s primary intention to increase the interaction of the user with the app together with the normative conception of health impacting the users’ understanding of their health stands in contrast to the self-constituting view on self-determination. Apps using a normative conception of health to increase user interaction impact the condition that the users’ wish to be healthy must be authentic. The app continuously confronts the user with their health status or their lack of health. The threshold at which the user perceives themselves as healthy is altered. Moreover, it is questionable if the user still has sufficient control over the course of their life if the objective of the app’s design is to create a certain dependency on the app. As we can see from the case of Jenny, the impact on the user’s conception of health can indeed determine the broader course of the user’s life. It is the case if the user engages with the app from a vulnerable position created by structural disadvantage.

Jenny uses the app from a disadvantaged position since the normative body image she perceives via social media has a negative impact on her mental health and well-being. This causes her to align her decision to use the app with the desire to live up to the unrealistic expectations regarding her physique, instead of following an internal authentic desire. Her disadvantage is not due to a specific agent but arises from different social practices and values concerning ideal body types that are reproduced in her social media feed, the design of the app as well as the app’s business model. In the hope of benefitting from the use of the app, she is confronted with the expectation to exercise more and transform her body due to the digital medical gaze that normatively charges her view on health. The increased risks of developing an eating disorder show how such normative aspects result in broader implications for the course of her life.

While trade-offs between health or expected health outcomes and self-determination are unproblematic for many users, they might be especially burdensome for disadvantaged individuals who already experience limitations to their well-being. Exposing groups of individuals who are subject to structural disadvantages to such value trade-offs constitutes a structural injustice itself.

## Personal Attachment

The trade-off between health benefits on one side and personal attachment on the other side occurs due to the risk of isolating the user from social interaction with their health professional if health services are substituted by mHealth products. The value of personal attachment is central to well-being and describes the need to form social relations with other human beings. Such relations include friendship and love as well as the feeling of belonging to a group of human beings and a sense of solidarity toward this group.

Loneliness has become a major public health concern in recent years (Lederman, 2021). For members of society struggling with loneliness and isolation, seeing a doctor might be a scarce opportunity to interact with another person and build a personal attachment that goes beyond a pure medical need and entails acknowledgment not just as a patient but as a whole person. This is by no means comparable to a friendship, but it can include a relationship of trust and companionship over many years. A doctor who is familiar with the patient’s life circumstances and medical history can have a more holistic understanding of the patient’s health status, too. By exchanging the service of a routine check-up or doctor’s appointment in person for a purely digital solution, the opportunity to create such a personal attachment entailing said acknowledgment might get lost.

Being isolated from others and unable to form such bonds can cause major mental health issues like anxiety or depression in the short term and post-traumatic stress disorder or prolonged grief symptoms in the long term leading to an increased risk of self-harm and suicide (Vrach and Tomar, 2020). Since especially the elderly are at risk of isolation, health care professionals, like doctors or nurses, might often be their only social contact or an important one among the few they have.

mHealth affects isolation in two ways. First, it can lead to increased isolation because doctor’s appointments are offered digitally. This forces people for whom such appointments are a rare opportunity for social contact to become even more isolated. Second, mHealth might streamline many processes making them more time efficient. This could have a positive effect on the available time of care workers which they can spend on individual patients who need more personal attention. Isolation and loneliness are nonetheless a risk likely to occur despite these cost savings. If the staff’s time is used more efficiently, the deployment of mHealth might result in a reduction of staff rather than providing the benefit of increased individual care to the patients.

Since mobile health apps increasingly find application in the professional care context. making the data from mHealth products available to one’s doctor contributes to the digital medical gaze the doctor has on the patient and therefore enforces viewing the patient less as a human being but rather as a collection of quantitatively measurable factors deviating from the norm. Due to the economic pressure on many hospitals, doctors have already less time to engage with their patients which necessarily results in focusing only on their most pressing medical needs rather than being able to acknowledge them as a complex human being that, aside from their condition, is situated in a socio-economic context and has mental and emotional states. Acknowledging this contextuality has severe implications for how care is delivered to the patient (Kidd and Carel, 2017), and thus the patient’s well-being.

This circumstance primarily impacts the dimension of equal respect although it has implications for personal attachment as well. Powers and Faden understand equal respect as ‘being recognized and treated as a moral being deserving of equal moral standing’ (Powers and Faden, 2019, p. 33). Patients who already belong to marginalized groups might feel especially mistreated if their precarious socio-economic position is neglected by their practitioner and they are not treated as equals. An obstacle to equal respect also diminishes the chances of building a personal attachment between practitioner and patient.

It is certainly not the obligation of a doctor to maintain a relationship with their patient and by this to contribute to their well-being. However, seeing the implementation of mobile health apps from the perspective of a public health intervention, they should be evaluated according to whether it prevents users from forming personal attachments. The ethical concern is based on the condition of wide implementation of public health apps, either incentivized as a preventative measure in the spirit of public health goals or introduced to primary as well as stationary care. Here it can be effective for patients or users as we can see in the case of John.

John uses the diabetes app in a situation of isolation he did not create himself but is the product of his social circumstances and public health developments that make it necessary or feasible for him. In the hope to benefit from the use, he must trade off some level of personal attachment for the health benefit. While this might not be a concern for users who are otherwise embedded in tight personal relationships with their friends and family, it adds to the already precarious situation of John and leaves him more disadvantaged than before. Of concern here is thus not only impairment in one core dimension of well-being but the interaction between the different dimensions of well-being since, for instance, loneliness can again lead to mental health issues which might result in a decreased ability to earn a living.

The loss of opportunities to form personal attachments is less of a problem for people who are socially situated in a way so they can still rely on their other personal attachments. Trading off this opportunity in exchange for a health benefit can, however, be more problematic for people who are already disadvantaged in a way that their opportunities to form personal attachments are scarce.

## Value Trade-offs and Disadvantage

The illustrated value trade-offs increase structural injustices for disadvantaged users. This is due to three main reasons. First, deprivations in well-being draw disadvantaged users of mHealth apps further away from the sufficiency threshold of well-being. Second, value trade-offs present the risk of impairments on the well-being of disadvantaged users. And third, value trade-offs can lead to an accumulative effect where deprivations in one dimension of well-being also lead to deprivations in another dimension.

The sufficientarian approach to social justice applied here, is rooted in the assumption that egalitarian theories of justice should not be concerned with distributing certain goods equally, but to ensure that everyone has ‘enough’(Frankfurt, 1987), thus securing a decent life for everyone. This approach is concerned with providing an answer to the question of what every human needs to have enough of. This naturally provokes the question of what is enough? Since the sufficientarian view on social justice relies on fundamentally important aspects of human life, like self-determination or personal attachment, and those aspects are ‘not commensurable in terms of any single quantitative standard’ (M. Nussbaum, 2007, p. 166), an adequate answer must refer to broader concepts like the value of a flourishing life allowing for a plurality of different aspects (Axelsen and Nielsen, 2017). For the present argument, the exact threshold of sufficiency is of less importance than its function in determining who should be the primary subject of considerations regarding the moral legitimacy of value trade-offs in mHealth apps.

Primarily disadvantaged persons, i.e. persons who are not capable of achieving the sufficiency threshold for leading a decent and flourishing human life, are central to this consideration. Like in the examples of Jenny and John they are subject to social circumstances, patterns of privilege, and disadvantages, presenting impairments to their well-being. People belonging to marginalized groups because of their age, gender, ethnicity, heritage, or physique among other factors, are vulnerable to value trade-offs because of their disadvantaged position. For those persons, having to deal with value trade-offs that impact what is fundamentally necessary to lead a decent human life, is unjust, because it draws them further away from the sufficiency threshold of well-being, and thus worsens their prospects. While users above the sufficiency threshold may be able to accept certain compromises to gain a health benefit, for those already disadvantaged every impact on their fundamental functionings turns them further away from a life in dignity. In the case of John, this means, that somebody who is already isolated to the degree that it severely impacts their well-being, is made even worse off due to the increase in isolation.

Furthermore, value trade-offs concerning the core dimensions of well-being can pose a risk of falling underneath a certain threshold of well-being. Being exposed to such a risk, although a sufficient level of well-being is realized for a person, constitutes a disadvantage, too (Wolff and De-Shalit, 2007, p. 72). In the case of Jenny, this means that somebody who in general is leading a decent life is exposed to the risk of developing severe impairments to their well-being when using a fitness tracking app. Again, the severity of the risk depends on the individual’s social circumstances and disposition.

Value trade-offs put the users at risk of being subject to an accumulative effect where deprivations in one dimension of well-being also affect other dimensions of their well-being. The clustering effect of disadvantage describes the accumulation of different kinds of disadvantages that are connected and run through various areas of an individual’s life (Wolff and De-Shalit, 2007). An educational disadvantage, for instance, can lead to poorer income which then results in a living situation compromising the individual’s health.

One might suggest that commercial mHealth apps offer a health benefit and thus contribute to reaching a sufficiently decent life. In the case of Jenny and John, however, the use of mHealth apps adds to their already high level of mental distress, negatively impacting their health. In Jenny’s case, developing an eating disorder can have long-term effects on her mental health and the insecurity about her body can affect how easily she engages in romantic relationships and forms personal attachments. In John’s case, isolation often causes distress and anxiety which also restricts his mental health.

Although users of mHealth apps must compromise dimensions of their well-being to some extent to gain the health benefit promised by the app, one could argue that the health benefit they gain positively affects other important dimensions of well-being. Interaction between different dimensions of well-being does not necessarily result in an increased accumulation of disadvantages but can have a fertilizing effect so that users might become more independent or self-determined. If this applies, the value trade-offs users must engage in are not unjust, even if the user engages with the app from a disadvantaged position.

For mHealth to enable a positive effect on various dimensions of well-being across different population groups it is necessary to mitigate the severity of the value trade-offs. This is especially true for populations not capable of reaching the sufficiency threshold for leading a life in dignity who should be entitled to the protection of fundamental conditions for well-being in the context of using mobile health apps. To comprehensively combat the structural injustices tied to deprivations of the users’ well-being, the focus on value trade-offs can provide a starting point to identify injustices that have not been discussed in the literature, yet.

If providers design products requiring disadvantaged persons to compromise their fundamental functionings to gain economic surplus, they create additional structural injustices. The deprivations of well-being for disadvantaged users resulting from value trade-offs are a structural injustice because they depend on circumstances outside the control of specific agents or entities. The roots of this problem lie not only in the design of the product or specific business models but also in the assumptions and expectations shaping the broader innovation processes of health technologies, respective policies, and the social practices defining the disadvantaged disposition of the users. What cannot be achieved through the altered design of mHealth apps, therefore, needs to be accomplished by policies that equal out the impairments to well-being experienced by the users and are directed at comprehensively promoting the fulfillment of the core dimensions of well-being.

## Conclusion

In this paper, I argued that users of commercial mHealth apps for self-monitoring must engage in value trade-offs for gaining the health benefit promised by the app. Those value trade-offs require users to compromise other fundamental dimensions of their well-being, like self-determination or personal attachment. Value trade-offs like this are instances of social practices harming disadvantaged populations because they present deprivations or risks to the sufficient fulfillment of multiple dimensions of well-being. Although users potentially gain in health, which is a central dimension of well-being, other dimensions are affected because the interaction between the dimensions can lead to a clustering effect of disadvantage. This is especially the case for self-determination and personal attachment. Therefore, value trade-offs increase disadvantage and present a type of structural injustice that depends on various social factors mirrored in the design and business model of many mHealth apps as well as the expectations and attitudes shaping its innovation and deployment processes.
